# Integrated Microbial Profiling Identifies Bacterial and Fungal Communities in Pseudomyxoma Peritonei Mucin

**DOI:** 10.1111/1751-7915.70457

**Published:** 2026-11-03

**Authors:** Rafaela Pezzopane, Marina Barbudo‐Lunar, Florina I. Bura, Melissa Granados‐Rodríguez, Ana Martínez‐López, Lidia Rodríguez‐Ortiz, Blanca Rufián‐Andújar, Javier López‐Jiménez, Verónica Luna‐Guerrero, José Alhama, Carmen Michán, Mari C. Vázquez‐Borrego, Álvaro Arjona‐Sánchez, Antonio Romero‐Ruiz

**Affiliations:** ^1^ Department of Biochemistry and Molecular Biology University of Cordoba Cordoba Spain; ^2^ Maimonides Biomedical Research Institute of Cordoba (IMIBIC) Cordoba Spain; ^3^ Pathology Unit Reina Sofía University Hospital Cordoba Spain; ^4^ Unit of Surgical Oncology, Surgery Department Reina Sofía University Hospital Cordoba Spain; ^5^ Institute of Marine Sciences of Andalusia (ICMAN‐CSIC) Cádiz Spain

**Keywords:** 18S rRNA sequencing, bacteriome, mycobiome, *Pseudomyxoma peritonei*, RNAscope, tumour microenvironment

## Abstract

Pseudomyxoma peritonei (PMP) is a rare mucinous malignancy in which tumour‐associated microbial communities remain poorly characterised, particularly, their fungal component and spatial organisation. We applied an integrated workflow combining 16S and 18S rRNA gene sequencing, microbial culture, histochemical staining, chromogenic RNAscope in situ hybridisation and a patient‐derived xenograft model to investigate bacterial and fungal communities in control tissues and low‐ and high‐grade PMP mucin. Bacterial and fungal DNA was detected in PMP‐associated mucin, with grade‐associated differences in bacterial composition and predicted functional potential. Viable bacteria were recovered from human PMP samples and xenograft‐derived mucin. Fungal structures were independently supported by periodic acid–Schiff and Calcofluor White staining, while RNAscope localised bacterial 16S and fungal 18S rRNA signals within epithelial and mucin‐rich tumour regions. Comparable microbial signals were observed in the xenograft model. Together, these complementary approaches identify PMP mucin as a multi‐kingdom microbial niche and provide the first characterisation of its associated mycobiome. This integrated analytical framework may facilitate the investigation of microorganisms in other low‐biomass, mucin‐rich tumour environments and provides a basis for future mechanistic studies of microbe‐host interactions in PMP.

## Graphical abstract

Integrated profiling of pseudomyxoma peritonei mucin revealed bacterial and fungal communities spatially supported by rRNA detection. Bacterial communities also displayed histological grade‐associated differences in predicted metabolic pathways, supporting PMP mucin as a complex multi‐kingdom microbial environment.

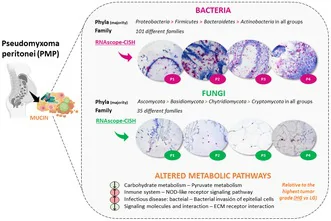

## 1 Introduction

Pseudomyxoma peritonei (PMP) is a rare mucinous disease characterised by progressive intraperitoneal accumulation of extracellular mucin, most commonly originating from ruptured appendiceal mucinous neoplasms (Carr et al. 2016). Histopathologically, PMP is classified into low‐ (LG) and high‐grade (HG) entities according to the Peritoneal Surface Oncology Group International (PSOGI) criteria, which correlate with prognosis and clinical behaviour (Carr et al. 2016). The incidence of PMP is estimated at 2–4 cases per million individuals per year, with a prevalence of approximately 25.1 cases per million (Smeenk et al. 2008).

Currently, the standard treatment strategy relies on an integrated CRS–HIPEC approach (Govaerts et al. 2020; Arjona‐Sanchez et al. 2021, 2026; Floriano et al. 2022). Although this strategy remains the most effective therapeutic option, it poses substantial clinical challenges. Tumour recurrence is common and contributes to significant disease‐related mortality (Mercier et al. 2019), indicating that key biological drivers of PMP persistence and progression remain poorly understood (Chua et al. 2012; Kusamura et al. 2021). Moreover, the extensive nature of CRS and the immunological impact of HIPEC, often combined with procedures such as splenectomy, increase the risk of postoperative immunosuppression and infectious complications (Govaerts et al. 2020; Arjona‐Sanchez et al. 2021; Floriano et al. 2022; Yang et al. 2023). These limitations highlight the need to better understand the biological mechanisms underlying PMP persistence and recurrence.

An emerging area of oncological research focuses on the role of microorganisms in cancer development and progression (Garrett 2015; Li et al. 2023; Cao et al. 2024). Microbial agents have been implicated in approximately 20% of human malignancies, influencing tumour growth, metastatic dissemination, inflammation and therapeutic response (de Martel et al. 2012; Elinav et al. 2013; Schwabe and Jobin 2013; Irrazábal et al. 2014; Louis et al. 2014; Sears and Garrett 2014). Recent studies have demonstrated that many solid tumours harbour an intratumoral microbiome, with microbial DNA and viable microorganisms across more than 30 distinct cancer types (Gagliani et al. 2014; Nejman et al. 2020; Kalaora et al. 2021). These findings challenge the long‐standing view of tumours as sterile environments and suggest that microorganisms may actively shape tumour biology, particularly, in cancers of the gastrointestinal and aerodigestive tract (Zepeda‐Rivera et al. 2024). The gut microbiota, for example, has been shown to interact with tumour‐associated mucins, alter host cell signalling and affect treatment response (Liu et al. 2022).

Beyond bacteria, increasing attention has been directed towards the tumour‐associated mycobiome. Fungal communities have been shown to modulate inflammation, immune responses and cancer progression through mechanisms involving complement activation, pattern‐recognition receptors and metabolic interactions with bacteria (Beheshti‐Maal et al. 2021; Dohlman et al. 2022; Narunsky‐Haziza et al. 2022). However, most studies of both the tumour microbiome and mycobiome rely primarily on DNA‐based sequencing approaches, which do not discriminate between viable, metabolically active microorganisms and residual or contaminating microbial material. This limitation is particularly relevant in low‐biomass tumours such as PMP, where distinguishing viable microorganisms from contaminating microbial DNA is essential.

PMP represents a unique and largely unexplored model to investigate tumour‐associated microbial ecosystems. This disease is characterised by extensive deposition of gel‐forming mucins, creating a mucin‐rich, hypoxic and relatively immune‐privileged environment that may function as a stable ecological niche for microorganisms. The appendiceal origin of most PMP cases further supports a potential link with intestinal microbiota and raises the possibility that microbial communities translocate and adapt to the peritoneal mucinous milieu. Consistent with this hypothesis, several studies have reported the presence of bacterial DNA and viable microorganisms in PMP‐associated mucin and peritoneal fluid (Gilbreath et al. 2013; Khamzina et al. 2022; Villarejo‐Campos et al. 2023; Nieciecki et al. 2025). Culture‐dependent and metagenomic analyses have identified taxa such as *Pseudomonas*, *Enterobacteriaceae*, *Bacteroides* and *Streptococcus*, among others, suggesting that these microorganisms may persist within the mucin‐rich environment and potentially contribute to chronic inflammation, mucin accumulation and tumour recurrence following CRS–HIPEC. Nevertheless, the taxonomic composition, functional potential, viability and spatial organisation of microbial communities in PMP remain poorly understood, while the fungal component has not been investigated. Recent analyses have therefore identified spatial characterisation of microbe–mucin–tumour interactions as an important priority for understanding PMP biology (Portela et al. 2025; Grant et al. 2026).

Therefore, the aim of this study was to establish an integrated analytical approach combining microbial sequencing, culture, histochemical detection and spatial RNA in situ hybridisation to characterise bacterial and fungal communities associated with PMP mucin and to explore grade‐associated differences in their taxonomic composition and predicted functional potential.

## 2 Experimental Procedures

### 2.1 Human Samples and Animal Models

The 15 samples analysed in this study were collected by the clinical staff of the Unit of Surgical Oncology at Reina Sofía University Hospital (HURS) (Cordoba, Spain). These samples were obtained from patients diagnosed with appendiceal‐origin PMP, who underwent complete CRS‐HIPEC. Patient diagnosis and classification adhered to the PSOGI classification guidelines.

Samples analysed include five from patients with LG PMP (LG1–LG5) and five from patients with HG PMP (HG1–HG5). Additionally, five control samples (C1–C5) were collected from non‐tumoral appendiceal tissue. These control samples were either from unaffected tissue of PMP patients or from individuals who had undergone prophylactic appendectomy for conditions unrelated to PMP. All patients were informed and signed the informed consent. Local Ethics Committee approved this study with number of reference S2300004.

Antibiotic prophylaxis administered intravenously prior to surgery varied among the groups: the control group (C) predominantly received two doses of 2 g of amoxicillin‐clavulanate before and during surgery; the LG PMP group received a single dose of 2 g of either amoxicillin‐clavulanate or cefazolin preoperatively and the HG PMP group generally received two doses of 2 g of amoxicillin‐clavulanate before and during surgery, with occasional administration of ciprofloxacin. All collected samples were aliquoted and stored at −80°C to preserve integrity. From sample collection during surgery to its handling in the laboratory, every effort was made to maintain sterility and minimise any potential contamination.

Additionally, we included PMP samples obtained from patient‐derived xenograft (PDX) mouse models. These PMP‐PDX models were developed using Nude (Foxn1nu) BALB/c mice and NSG mice (NOD.CB17/AlhnRj‐Prkdcscid, Janvier Labs), aged 6–9 weeks and maintained on a standard chow diet. All mice were bred and housed in a specific pathogen‐free barrier facility at the Maimonides Institute of Biomedical Research of Cordoba (IMIBIC), with ad libitum access to food and water. The timeframe from PMP transplantation to the collection of tumour samples in mice ranged from 25 to 80 days, depending on the PMP‐PDX line. Antibiotics prophylaxis was not performed in mice.

All animal procedures were approved by the Ethical Committee for animal experimentation of the University of Cordoba. Additionally, authorisation was granted by the Ministry of Agriculture for the development of preclinical models (certificate number 25/08/2025/080). This study adhered to the regulatory standards, with human sample collection approved by the HURS ethics committee and conducted with informed consent of the patients. Data handling adhered to the Spanish Data Protection Law (LOPD, BOE No. 294, 6 December 2018).

### 2.2 DNA Extraction, Quantification and Sequencing

Microbial cells from the tissues were enriched using a pre‐treatment protocol designed to degrade mucin prior to DNA extraction for the release of encapsulated microorganisms. For each sample, 3 mg of dithiothreitol (DTT) was dissolved in 1 mL of 100 mM Tris–HCl buffer solution at pH 7.5, resulting in a final DTT concentration of 20 mM. This solution was then added to 50 mg of each sample, after which all samples were incubated at 30°C with agitation at 300 rpm for 30 min. To enhance the degradation process, samples were gently resuspended by pipetting every 5 min. Samples were centrifuged at 15,000*g* at 4°C for 10 min to remove residual mucin. The supernatant, containing mucin remnants, was carefully discarded, while the resulting pellet containing microbial cells was preserved on ice until DNA isolation was performed.

The microbial DNA of the PMP samples was extracted using the ZymoBIOMICS DNA Miniprep Kit (Zymo Research). DNA extraction was carried out following the manufacturer's instructions, with minor modifications. Specifically, cell lysis was performed by adding 750 μL of DNA/RNA Shield (Zymo Research) to microbial pellets obtained after mucin degradation. This buffer was selected in place of the kit's standard lysis solution due to its superior nucleic acid preservation performance, as reported by the manufacturer. DNA was eluted in 50 μL of DNase/RNase‐free water and stored at −30°C. DNA concentration was quantified via spectrophotometry. Standard PCR using 16S and 18S rRNA genes primers was performed to confirm the presence of bacterial and fungal DNA, respectively and to assess the presence of potential PCR inhibitors. The sequences of the primers used were as follows: 16S (Forward: 5′‐TGGTGGAATTTCCTGTGTAGCGGTGAA‐3′; Reverse: 5′‐AAGGCCAGGTAAGGTTCTTCGCGTTGC‐3′); 18S (Forward: 5′‐TTTGAGGCAATAACAGGT‐3′; Reverse: 5′‐ANCCATTCAATCGGTANT‐3′). For 16S, amplicons were generated and sequenced from genomic DNA at the Genomic Unit of the Central Services for Analysis (SCAI) of the University of Cordoba (Spain); for 18S, the unit performed sequencing of the provided amplicons, both using the Ion S5 System (Thermo Fisher Scientific).

### 2.3 Metabarcoding Analysis: Taxonomic Profiling, Diversity Metrics and Functional Characterisation of Bacterial Communities

For 16S rRNA gene analysis, the Ion 16S Metagenomics Kit (Thermo Fisher Scientific) and the Ion Reporter 5.0 software were used. This kit enables the amplification and sequencing of multiple 16S rRNA gene hypervariable regions (V2, V3, V4, V6–7, V8 and V9). The V3 region primer pair was selected for further analysis due to its highest number of total identifications and greatest taxonomic diversity.

For 18S rRNA gene analysis, the FASTQ files were analysed using the QIIME 2 workflow (2024.5 version). To do so, the primer sequences were trimmed from the read sequences using the *Split_on_Primer.py* script. Subsequently, these reads were denoised into amplicon sequence variants (ASVs) using the DADA2 pipeline incorporated in QIIME 2. Taxonomical classification was performed using a Naive Bayes classifier trained on the sequenced 18S region. For the creation of the reference database, preformatted SILVA files provided by QIIME 2 (version 138) were used, retaining only the sequences matching our primer pair (Bolyen et al. 2019).

Alpha diversity was evaluated at the family level using Pielou's evenness, Shannon diversity index and the Gini–Simpson index, which together capture both species richness and evenness within microbial communities (Shannon 1948; Simpson 1949; Pielou 1966). Beta diversity was computed to assess differences in community composition between samples. The parameters and statistical methods applied for diversity calculations are described in detail in the statistical analysis section.

Functional pathway analysis was performed using Tax4Fun (Aßhauer et al. 2015), based on the Kyoto Encyclopaedia of Genes and Genomes (KEGG) database, using 16S rRNA gene sequencing data obtained from all samples.

### 2.4 Statistical Analysis

Statistical analyses using R programming language v4.3 were performed to discriminate among the three conditions analysed (C, LG and HG). Additionally, we used Bioconductor R packages vegan v2.6‐10 and mia v1.12.0. The normality of the data distribution was evaluated by Shapiro–Wilk test (GraphPad Prism 8.0.2) and the normality assumptions were not met. A centred log‐ratio (clr) transformation was applied to the data. The differences in abundance of bacterial taxa were evaluated for phylum, family, genus and species using the Kruskal–Wallis non‐parametric test. To mitigate the risk of false positives due to multiple comparisons, we applied the Benjamini–Hochberg (BH) method with significance set at an adjusted *p* < 0.05.

Alpha diversity across the experimental conditions (C, LG and HG) was evaluated at the family level using two standard indices calculated from Ion Reporter (bacteriome) and QIIME (mycobiome) raw counts. To assess statistical differences between groups, the Kruskal–Wallis test was applied to these computed indices, providing a robust non‐parametric comparison of taxonomic richness and evenness within the samples. To assess beta diversity, multidimensional scaling (MDS) analysis based on Aitchison distance was performed at the family level. Beta diversity was tested using PERMANOVA (permutational multivariate analysis of variance) with transformed counts (centred log‐ratio transformation). PERMANOVA does not require the data to be normally distributed and was performed with 9999 permutations and corrected using the BH method (< 5% FDR). The same pipeline was implemented for the 18S rDNA gene analyses to ensure consistency in methodology.

The predicted bacterial metabolic pathways obtained from Tax4Fun (presented as relative abundances) were also subjected to the same statistical analysis. We employed the Kruskal–Wallis non‐parametric test to assess significant differences in these pathways, followed by Benjamini–Hochberg (BH) correction for multiple comparisons. Pathways with an adjusted *p*‐value < 0.05 were considered statistically significant. All graphical representations were generated using GraphPad Prism 8.0.2 and the ggplot2 3.5.2 R package.

### 2.5 Microorganisms Culture

For the culture of microorganisms, fresh untreated mucin samples and mucin treated with 1 M NaOH were used. The NaOH treatment was used to disaggregate mucus and enhance cell exposure to the culture medium.

Due to the diversity of microorganisms identified via metabarcoding and the challenges in replicating optimal growth conditions, different media and cultivation conditions were employed. Luria–Bertani (LB) and yeast peptone dextrose (YPD) media were used for bacterial and fungal identification, respectively. To simulate the low‐oxygen environment of tumour growth, anaerobic media such as Fastidious Anaerobe Agar (FAA) with Horse Blood (Thermo Scientific) and Schaedler Agar with Sheep Blood (Thermo Scientific) were also used.

Samples were inoculated onto Petri dishes with different solid media. Plates for aerobic growth were incubated at 30°C and 37°C for 3–5 days. For anaerobic growth, an Anaerogen GasPack Generated System (Oxoid, Thermo Fisher Scientific) 2.5 L was used at 37°C for a minimum of 7 days.

Liquid cultures in LB and YPD were incubated under agitation at 30°C and 37°C. Samples showing signs of growth were subsequently streaked onto Petri dishes with solid media, under the same growth conditions mentioned above, to obtain isolated colonies.

To prevent environmental contamination, aseptic techniques were maintained throughout the experimental procedures. Furthermore, negative controls were included for each microbiological culture batch.

Microorganisms were identified from pure colonies collected during the exponential growth phase using MALDI‐TOF mass spectrometry (MALDI Biotyper system, Bruker). The isolated colonies were first suspended in 300 μL of sterile water and then subjected to an extraction protocol by adding 900 μL of pure ethanol. Following cell lysis with formic acid and acetonitrile, the extracted ribosomal proteins were deposited onto a MALDI plate and overlaid with an *α*‐cyano‐4‐hydroxycinnamic acid matrix. Samples were analysed using a MALDI‐TOF/TOF Ultraflextreme system (Bruker) and the resulting spectra were compared against the MBT Compass Library database (> 7000 strains) for identification at the genus and species levels.

When MALDI‐TOF/TOF failed to identify the species, Sanger sequencing was performed. In this case, the colony was resuspended in distilled water and homogenised. The sample underwent thermal lysis in a water bath at 100°C for 10 min, followed by cooling on ice and centrifugation at maximum speed. The resulting supernatant served as the DNA template for a PCR targeting the 16S region, using standard primers and optimised reaction conditions. PCR products were visualised on 1% agarose gels, purified and submitted for Sanger sequencing to the Genomics Unit of SCAI.

### 2.6 Fungal Staining and Microscopy Procedures

In addition to the metabarcoding analysis of the samples, two staining techniques, Calcofluor White (CFW) and periodic acid–Schiff (PAS), were employed to evaluate the presence of fungal structures in various biological specimens. CFW at 0.1% (Fluka, 18,909) was used as a fluorescent dye to detect chitin in fungal cell walls. Although not entirely specific to fungi, CFW binds strongly to chitin and cellulose, allowing visualisation under UV or blue light excitation (Restrepo et al. 2024). Fluorescent images were acquired using a Leica THUNDER Imager 3D Assay fluorescence microscope and processed with ImageJ software (v. 1.54).

Formalin‐fixed, paraffin‐embedded (FFPE) sections of mucinous tumour tissue were first heated at 60°C on a slide warmer for 30 min. The slides were then deparaffinised by washing them three times in xylene for 5 min each. This was followed by sequential ethanol washes: once in 100% ethanol, twice in 95% ethanol and twice in 70% ethanol, each for 3 min. The slides were then rinsed in distilled water for 3 min. A drop of 0.1% CFW was applied to each section, covered with a coverslip and incubated for 1–2 min. Visualisation was performed under UV or blue light excitation (*λ*_Ex_ = 355 nm) at ×400 magnification. To analyse liquid mucin samples, approximately 250 μL of the sample was mixed with 50 μL of 0.1% CFW. After thorough mixing, 50 μL of the stained suspension was transferred to a microscope slide, covered with a coverslip and incubated for 1 min before imaging under the same fluorescence settings.

In parallel, PAS staining was performed on slides containing sections of mucinous tumour tissue to detect polysaccharides in fungal cell walls and extracellular matrices. This technique was performed by the Pathology Unit at Reina Sofía University Hospital (Cordoba, Spain). Briefly, FFPE sections were deparaffinised and rehydrated using the same xylene and ethanol series described above. Sections were then oxidised in 1% periodic acid for 5 min, rinsed in distilled water and incubated with Schiff's reagent for 15 min in the dark. After thorough washing in running tap water for 5–10 min to develop the magenta coloration, sections were counterstained with haematoxylin, rinsed, dehydrated through graded alcohols, cleared in xylene and mounted with coverslips. Samples were evaluated under bright‐field Leica DM2000 LED microscopy at ×40 magnification to assess the presence and distribution of PAS‐positive fungal structures.

### 2.7 RNAscope In Situ Hybridisation

The spatial presence of bacterial and fungal ribosomal RNA was evaluated using RNAscope 2.0 single‐plex chromogenic kit (Advanced Cell Diagnostics Inc. Hayward, CA) (Wang et al. 2012). Probes specific to microbial ribosomal RNA targets for 16S rRNA gene (RNAscope Probe‐EB‐16SrRNA, Cat No. 464461) and for fungal 18S rRNA gene (RNAscope Probe‐Fungus‐18SrRNA‐O1, Cat No. 431891) were used. The assay was optimised and performed according to the manufacturer's instructions. Briefly, 5 μm sections of FFPE specimen were cut and placed on SuperFrost Plus charged glass slides, after which they were deparaffinised in a series of xylene and 100% ethanol steps. The deparaffinised slides were then subjected to a series of pre‐treatment steps before progressing onto hybridisation with target probes. This involved placing approximately 140 μL of the individual target probes from a ready‐to‐use bottle onto the slide, ensuring full coverage of the tissue sections. To validate assay performance and RNA integrity, a positive control probe targeting the human housekeeping gene PPIB (RNAscope Positive Control Probe—Hs‐PPIB‐O1, Cat No. 313901) and a negative control probe targeting the bacterial gene dapB (RNAscope Negative Control Probe‐DapB, Cat No. 310043) were included. Probe hybridisation was performed in all cases at 40°C for 2 h in a HybEZ oven, followed by a series of six signal amplification steps (AMP1–AMP6) using RNAscope 2.5 HD Detection Reagents (Red). Signal detection was achieved using a Fast Red chromogen, with the incubation time adjusted according to the type of probe used. The slides were then counterstained with 50% Gill's haematoxylin, blued with ammonium hydroxide and mounted using EcoMount mounting medium. All slides were processed in parallel under identical conditions. Specific punctate red signals were interpreted as positive microbial RNA detection, whereas negative control slides showed no such signal. The absence of signal in negative controls and the robust signal in positive control probes confirmed assay specificity and tissue RNA preservation.

## 3 Results

### 3.1 Bacterial Community Composition Differs Across Control, LG and HG PMP Samples

To characterise bacterial community dynamics during PMP progression, 16S rRNA gene sequencing was performed on control appendiceal tissues (C), LG and HG PMP samples. Bacterial DNA was detected in all experimental groups, confirming the presence of bacterial communities in both normal appendiceal tissue and tumour‐associated mucin. Although the average number of bacterial identifications tended to decrease from controls to HG tumours, these differences did not reach statistical significance (Figure 1A).

**FIGURE 1:**
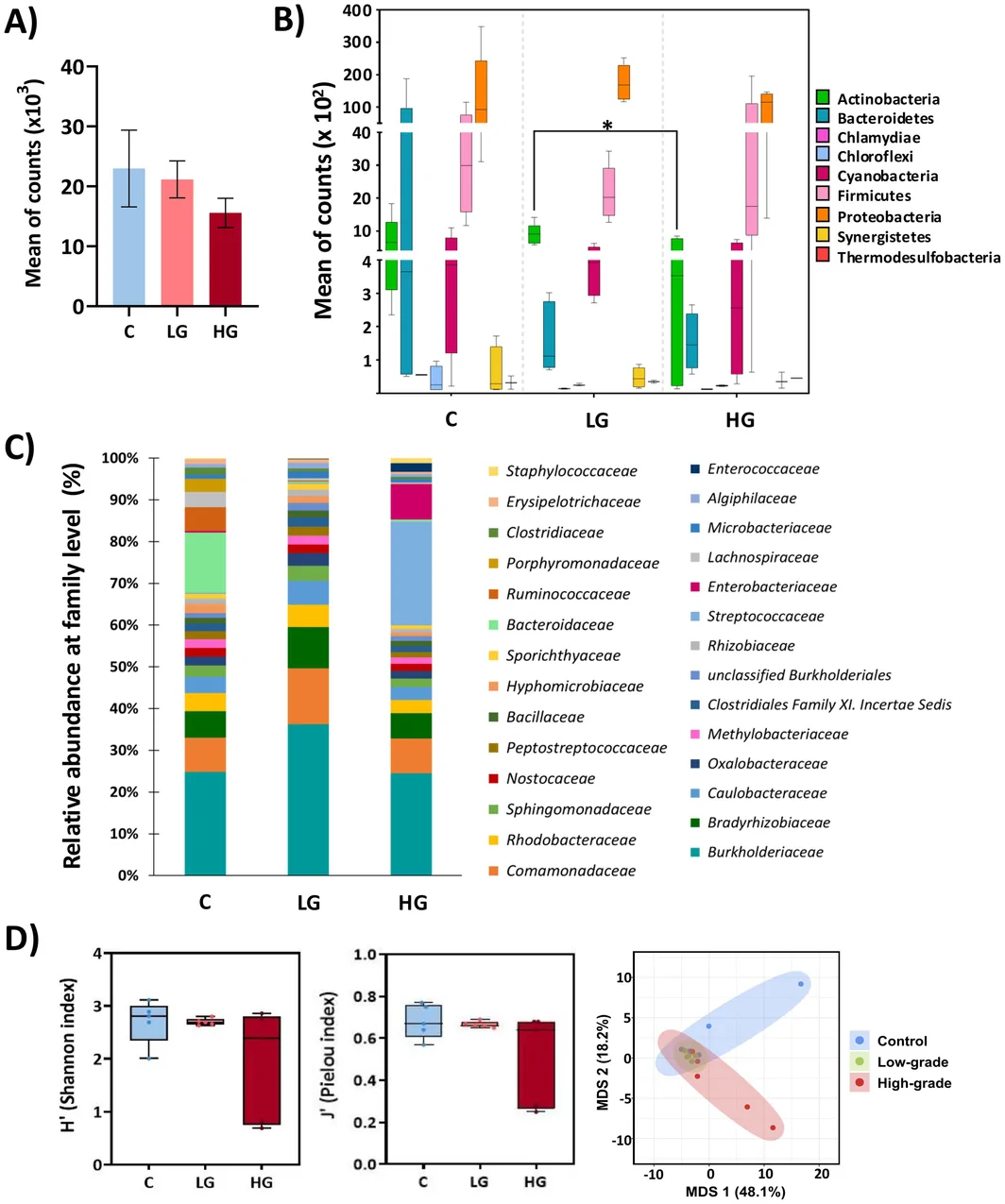
Bacterial community composition and diversity across control samples and PMP mucinous tissue samples. (A) Total number of 16S rRNA gene sequences detected per sample in control appendiceal tissue (C), low‐grade PMP mucinous samples (LG) and high‐grade PMP mucinous samples (HG). (B) Relative abundance of bacterial phyla based on 16S rRNA sequencing in C, LG and HG samples. A significant difference was observed between groups (*p = 0.017). (C) Relative abundance of bacterial families (≥ 1% in at least one group). (D) Alpha diversity indices (Shannon–Wiener and Pielou's evenness) and beta diversity analysis (MDS based on Aitchison distance) of bacterial communities in C, LG and HG samples.

Taxonomic profiling revealed grade‐associated differences in bacterial community composition across disease stages. At the phylum level, Firmicutes, Bacteroidetes and Proteobacteria predominated in all groups, whereas Actinobacteria differed significantly between LG and HG PMP samples (adjusted *p* = 0.017) (Figure 1B). At the family level, control tissues were enriched in dominant commensal bacterial families, including Lachnospiraceae, Ruminococcaceae and Bacteroidaceae. In contrast, PMP samples, particularly HG tumours, showed a relative reduction in these families together with an increased representation of Enterobacteriaceae and Streptococcaceae, consistent with progressive compositional remodelling of the tumour‐associated bacteriome (Figure 1C).

Alpha‐diversity analyses showed no significant differences in Shannon diversity or Pielou's evenness among groups. However, HG samples displayed greater inter‐individual variability than controls and LG tumours, indicating increased community heterogeneity across histological groups. Similarly, MSD based on Aitchison distances revealed partial overlap between control and LG samples, whereas HG samples tended to segregate from the remaining groups despite the absence of statistically significant differences by PERMANOVA (*F* = 1.47, *R* ^2^ = 0.20, *p* = 0.09) (Figure 1D).

Complementary visual analyses further supported these findings. Krona plots provided a qualitative overview of the progressive remodelling of bacterial community composition across disease stages and illustrated the greater heterogeneity observed among HG samples (Figure S1).

Collectively, these findings indicate that PMP progression is accompanied by progressive remodelling of the tumour‐associated bacteriome, characterised by changes in community composition and increased inter‐individual heterogeneity, particularly in HG disease.

### 3.2 Culture‐Based Isolation Confirms the Presence of Viable Bacteria in Human PMP and the PDX Model

To determine whether the bacterial communities identified by 16S rRNA gene sequencing represented viable microorganisms, culture‐based microbiological analyses were performed on freshly collected PMP specimens and PDX tumours. Viable bacteria were recovered from the majority of human PMP samples and from all analysed PDX tumours, demonstrating that tumour‐associated bacteria remain viable within the mucin‐rich microenvironment and can be maintained following xenotransplantation. Representative bacterial growth from human PMP samples and the corresponding PDX models is shown in Figure S2 and Table 1.

**TABLE 1:** Cultured bacterial isolates from PMP mucin and comparison with taxonomic assignments obtained by 16S rRNA gene sequencing.

Culture	16S rRNA genes sequencing			
	Order	Family	Genus	Species
*Sphingomonas paucimobilis* ^a^, ^b^	+	+	+	−
*Microbacterium laevaniformans* ^a^	+	+	+	−
*Leifsonia* sp.^a^	+	+	−	−
*Paenibacillus pabuli* ^a^	+	−	−	−
*Staphylococcus capitis* ^a^	+	+	+	−
*Lachnotalea glycerini* ^a^	+	+	−	−
*Staphylococcus warneri* ^a^	+	+	−	−
*Bacillus amyloliquefaciens* ^b^	+	+	+	−
*Bacillus licheniformis* ^a^	+	+	+	−
*Escherichia coli* ^b^	+	+	+	+
*Enterococcus gallinarum* ^a^	+	+	+	−
*Staphylococcusaominis* ^a^	+	+	+	+
*Staphylococcus xylosus* ^b^	+	+	+	−
*Parabacteroides goldsteinii* ^b^	+	+	+	+
*Enterococcus faecalis* ^a^	+	+	+	+
*Lactobacillus johnsonii* ^a^	+	+	+	−
*Phocaeicola vulgatus* ^a^	+	+	+	+
*Cutibacterium acnes* ^b^	+	+	+	+

^a^ Species isolate from human PMP mucinous samples.

^b^ Species identified from PMP‐PDX mucinous samples.

Culture‐based analyses recovered a diverse range of bacterial taxa, including members of the genera *Staphylococcus*, *Enterococcus*, *Bacillus*, *Escherichia*, *Parabacteroides*, *Phocaeicola* and *Sphingomonas*, among others (Table 1). Taxonomic assignments obtained from cultured isolates were consistent with the 16S rRNA gene sequencing analysis at different taxonomic levels, providing complementary evidence that at least part of the PMP‐associated bacteriome comprises viable microorganisms. Specifically, *Sphingomonas paucimobilis* was identified at the species level through a culture‐based analysis, while 16S rRNA gene sequencing detected *Sphingomonas* at the genus level but did not allow the taxonomic classification to be determined at the species level. Representative bacterial cultures obtained from human PMP samples and the corresponding PDX models are shown in Figure S2.

### 3.3 First Identification of a Fungal Microbiome in Pseudomyxoma Peritonei

Because the fungal component of the PMP‐associated microbiome has not previously been investigated, we performed 18S rRNA gene sequencing on control appendiceal tissues (C), LG and HG PMP samples. Fungal DNA was detected in all experimental groups, providing, to our knowledge, the first evidence of a fungal microbiome associated with PMP. The mean number of fungal identifications showed a tendency to increase in PMP samples, particularly in HG tumours, although these differences did not reach statistical significance (Figure 2A).

**FIGURE 2:**
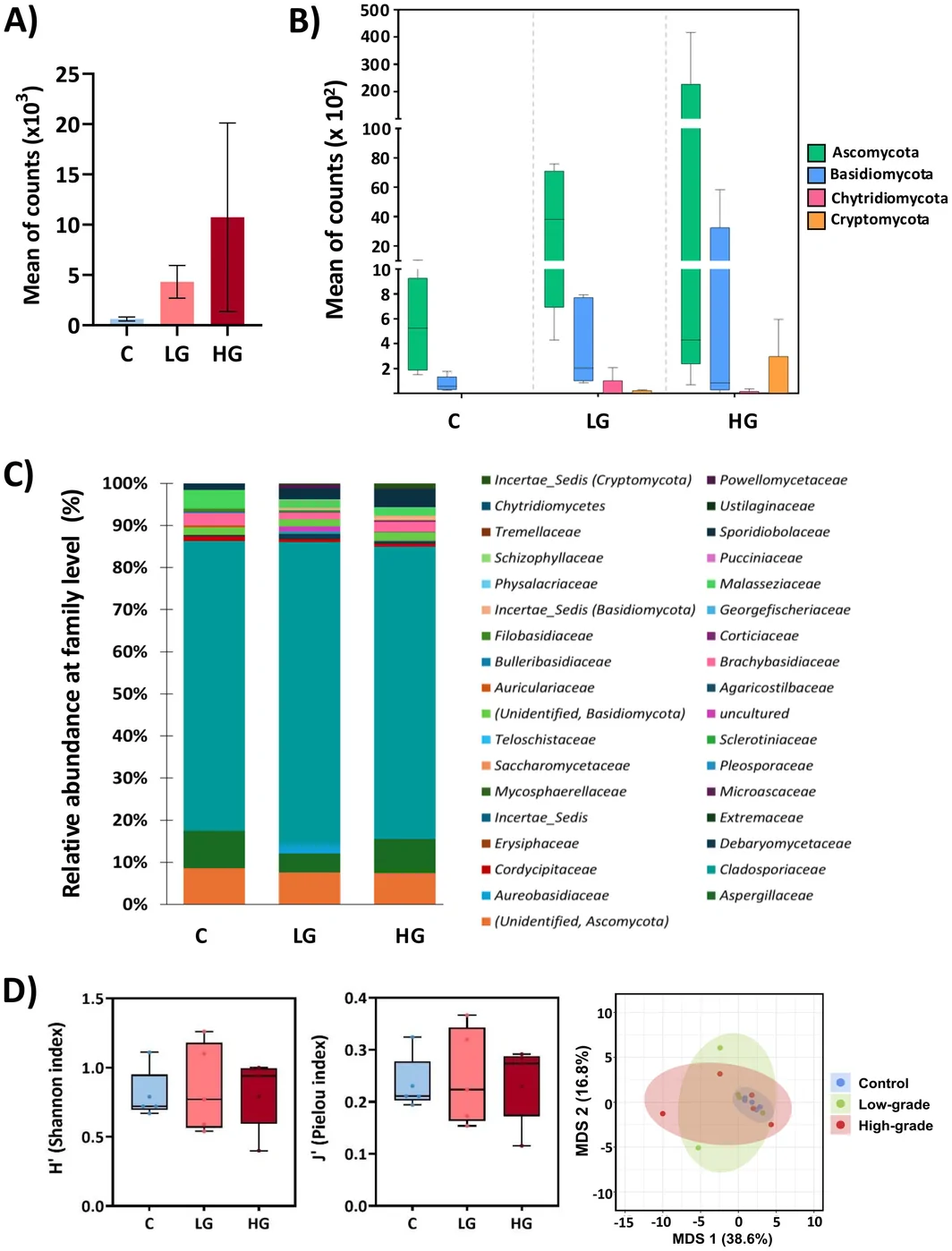
Fungal community composition and diversity across control and PMP mucinous samples. (A) Total number of 18S rRNA gene sequences detected per sample in control appendiceal tissue (C), low‐grade PMP mucinous samples (LG) and high‐grade PMP mucinous samples (HG). (B) Relative abundance of fungal phyla based on 18S rRNA sequencing in C, LG and HG samples. (C) Relative abundance of fungal families identified by 18S rRNA gene sequencing (≥ 1% in at least one group). (D) Alpha diversity indices (Shannon–Wiener and Pielou's evenness) and beta diversity analysis (MDS based on Aitchison distance) of fungal communities in C, LG and HG samples.

Taxonomic profiling revealed that fungal communities were dominated by Ascomycota across all experimental groups, followed by Basidiomycota, whereas Chytridiomycota and Cryptomycota were detected at much lower relative abundances (Figure 2B). At the family level, fungal community composition remained broadly comparable among control, LG and HG samples, with Cladosporiaceae representing the predominant family together with several lower‐abundance taxa, including Aspergillaceae (Figure 2C).

Alpha‐diversity analyses showed no significant differences in Shannon diversity or Pielou's evenness among the experimental groups. Likewise, MSD based on Aitchison distances demonstrated substantial overlap between control, LG and HG samples, indicating that the overall structure of the fungal community remained broadly conserved across disease stages (Figure 2D). Complementary Krona visualisations provided a qualitative overview of the fungal communities and supported the presence of broadly comparable taxonomic profiles across individual samples (Figure S3).

Collectively, these findings provide the first evidence that PMP harbours a fungal microbiome, establishing fungi as a previously unrecognised component of the PMP tumour microenvironment. Although fungal DNA was detected in both control and PMP tissues, tumour‐associated mucin showed a clear tendency towards increased fungal burden while maintaining a broadly conserved community structure.

### 3.4 Histological Validation of Fungal Structures Within PMP‐Associated Mucin

To determine whether the fungal DNA detected by 18S rRNA gene sequencing corresponded to fungal structures within PMP tissues, histochemical analyses were performed on tumour‐associated mucin using PAS and CFW staining.

PAS staining revealed fungal structures embedded within the extracellular mucin of both LG and HG PMP samples (Figure 3A–C). Because PAS also stains host polysaccharides, CFW staining was subsequently used to specifically detect chitin‐containing fungal cell walls. Calcofluor fluorescence confirmed the presence of discrete fungal structures within the tumour‐associated mucin, providing independent histological evidence supporting the presence of fungi in PMP (Figure 3A–C).

**FIGURE 3:**
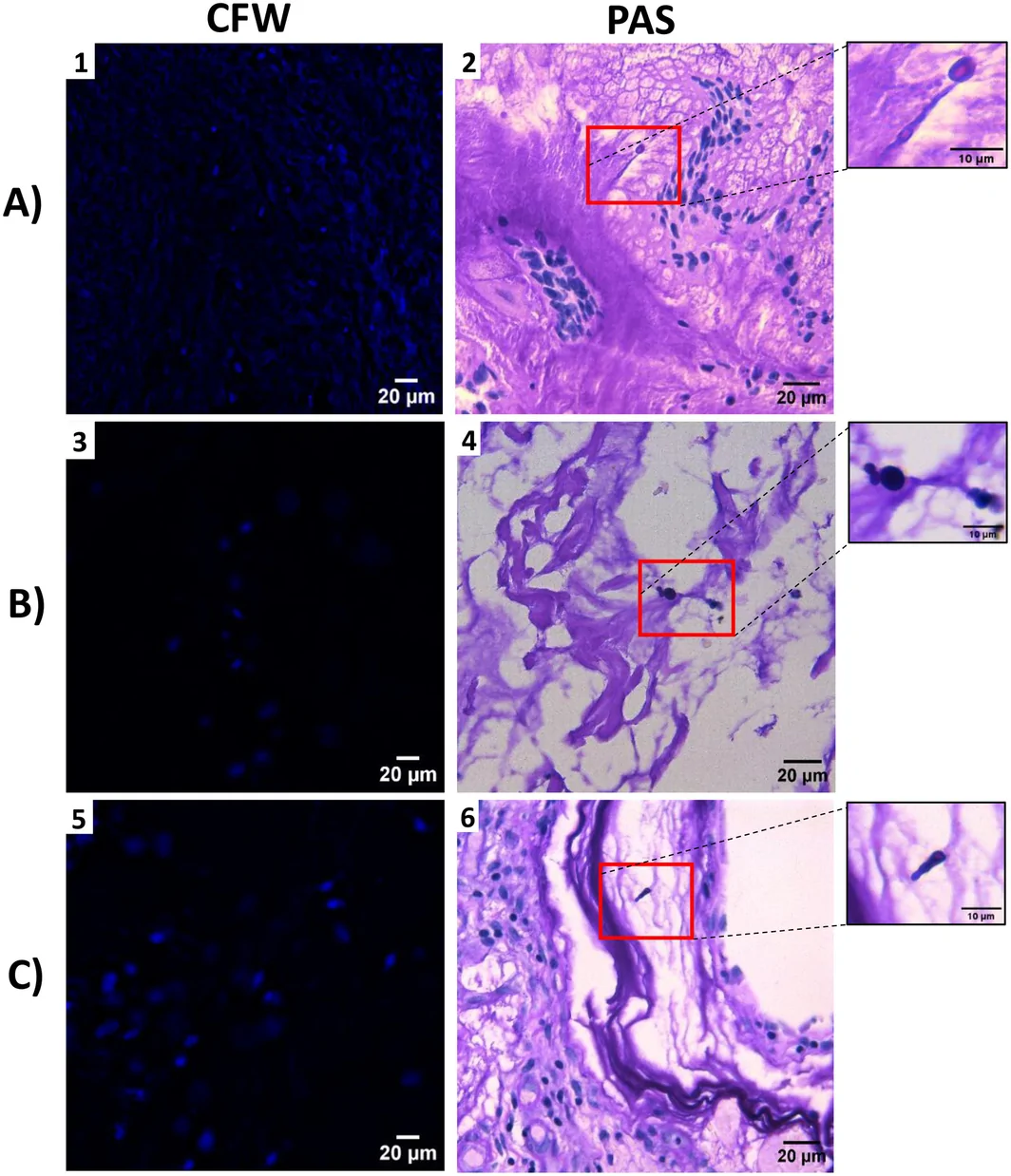
Histological and fluorescence‐based visualisation of fungal structures in PMP mucinous samples. (A–C) Representative images from three independent PMP patients showing fungal elements detected by Calcofluor White (CFW, 0.1%) and periodic acid–Schiff (PAS) staining. Left panels correspond to CFW fluorescence, highlighting chitin‐rich fungal cell walls, while right panels show PAS‐stained histological sections of mucinous tissue. Magnified insets in PAS images indicate round, spore‐like fungal structures embedded within the mucin matrix. CFW images were acquired at ×400 magnification. Samples include both formalin‐fixed paraffin‐embedded tissue sections (A1) and liquid mucin preparations (B3 and C5). See Methods for experimental details.

Collectively, these findings provide complementary histological evidence supporting the presence of fungal structures within PMP‐associated mucin and independently validate the fungal DNA detected by 18S rRNA gene sequencing.

### 3.5 RNAscope Confirms the Spatial Localisation of Bacterial and Fungal rRNA in PMP Tissues

To determine the spatial distribution of tumour‐associated microorganisms within PMP tissues, chromogenic RNAscope in situ hybridisation was performed using probes targeting bacterial 16S and fungal 18S rRNA.

Distinct punctate signals corresponding to bacterial 16S rRNA were detected within epithelial and mucin‐rich tumour regions in all analysed PMP samples, demonstrating the spatial localisation of bacteria within the tumour microenvironment (Figure 4A). Similarly, fungal 18S rRNA signals were identified within tumour‐associated mucin, providing direct spatial evidence supporting the presence of fungal microorganisms in PMP tissues (Figure 4B). The specificity of the assay was confirmed using the positive control probe (Hs‐PPIB), which produced the expected hybridisation signal, whereas no signal was observed with the negative control probe (DapB) (Figure 4C).

**FIGURE 4:**
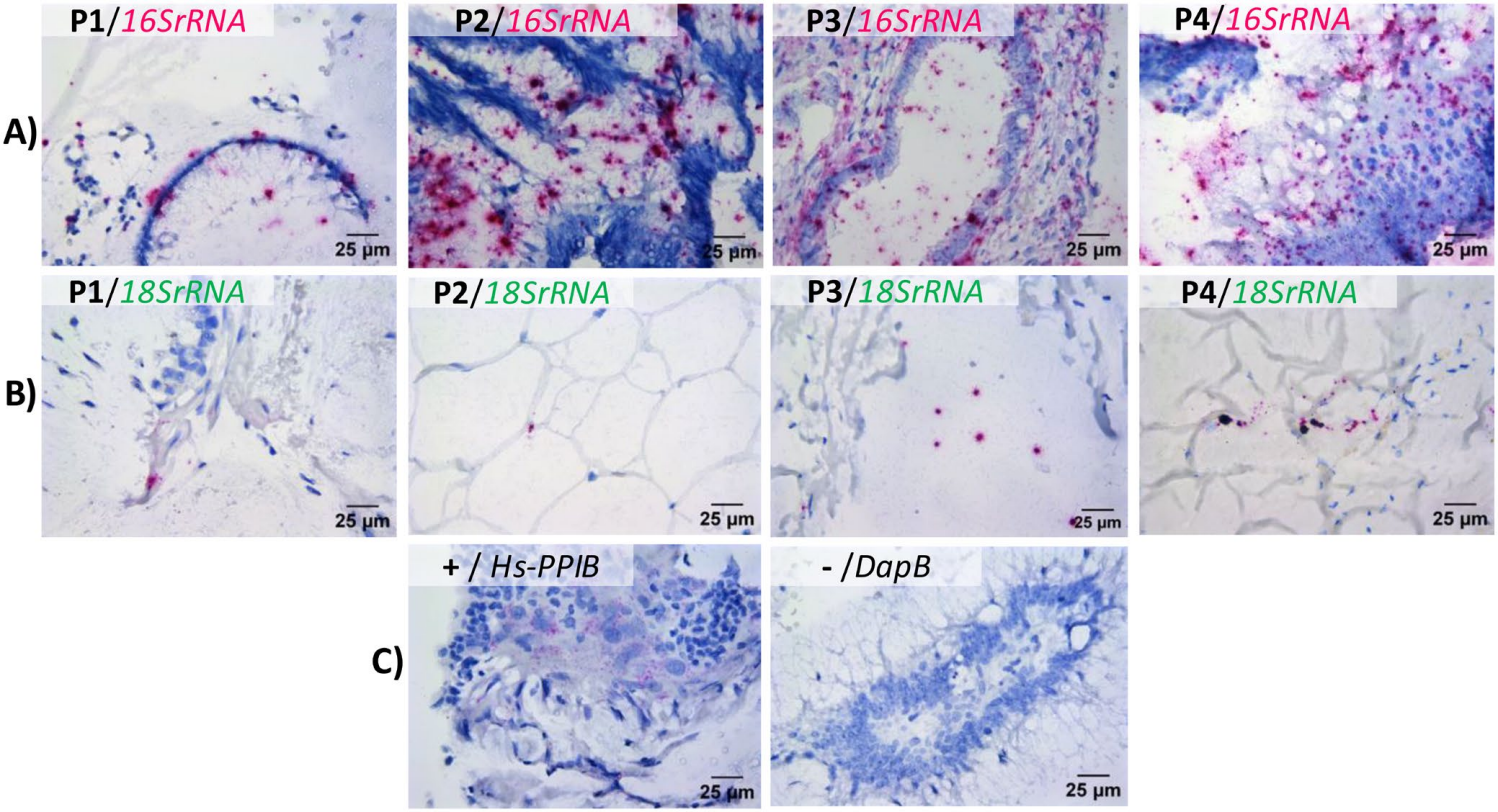
Detection of bacterial and fungal rRNA in PMP tissues by RNAscope chromogenic in situ hybridisation (RNAscope‐CISH). Formalin‐fixed, paraffin‐embedded (FFPE) PMP tissue sections (4–5 μm) containing epithelial and mucin‐rich regions were analysed. Representative detection of bacterial 16S rRNA (A) and fungal 18S rRNA (B) in PMP samples from different patients (P1–P4). Red punctate signals correspond to individual amplified rRNA molecules; increased signal density and clustering indicate higher transcript abundance. Microbial rRNA signals are observed within both epithelial and mucinous tumour compartments. (C) Assay controls: positive control using a probe targeting the human housekeeping gene Hs‐PPIB, confirming RNA integrity and assay performance and negative control using the bacterial DapB probe, showing absence of detectable signal and indicating lack of nonspecific hybridisation. Sections were counterstained with haematoxylin and blued with ammonium hydroxide. Scale bar = 25 μm. Original magnification ×40.

Comparable bacterial 16S and fungal 18S rRNA signals were also detected in PMP PDX tumours, indicating that the spatial association of microbial communities with tumour‐associated mucin was maintained following xenotransplantation (Figure S4).

Collectively, these findings provide direct spatial evidence of bacterial and fungal rRNA within the PMP tumour microenvironment in both human tissues and the corresponding PDX model, independently validating the sequencing, culture‐based and histological findings and providing convergent evidence that PMP harbours a tumour‐associated multi‐kingdom microbiome.

### 3.6 Predicted Functional Differences Between LG and HG PMP‐Associated Bacteriomes

To investigate whether bacterial communities associated with different PMP grades also differed in their predicted functional potential, pathway enrichment analysis was performed using Tax4Fun based on 16S rRNA gene sequencing data.

No significant differences in predicted functional pathways were identified between control tissues and PMP samples after correction for multiple comparisons. In contrast, significant differences in predicted functional profiles were detected between bacterial communities associated with LG and HG PMP (Figure 5; Table S1).

**FIGURE 5:**
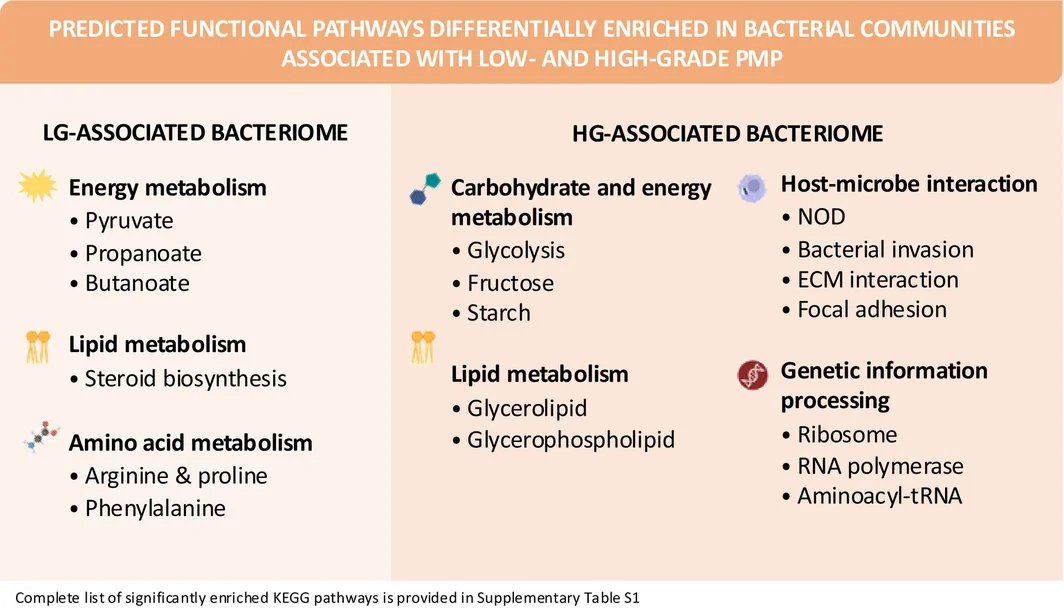
Predicted functional pathways differentially enriched in bacterial communities associated with low‐ (LG) and high‐grade (HG) pseudomyxoma peritonei. Functional prediction was performed using Tax4Fun based on 16S rRNA gene sequencing data. Only representative KEGG pathways showing significant differences after Benjamini–Hochberg correction (adjusted *p* < 0.05) are shown. Compared with LG‐associated bacteriomes, HG‐associated bacterial communities were enriched in pathways related to carbohydrate and energy metabolism, lipid metabolism, host–microbe interactions and genetic information processing. Conversely, LG‐associated bacteriomes showed enrichment of pathways involved in energy metabolism, lipid metabolism and amino acid metabolism. The complete list of significantly enriched pathways is provided in Table S1.

Compared with LG‐associated bacteriomes, HG‐associated bacterial communities showed significant predicted enrichment of pathways related to carbohydrate and energy metabolism, lipid metabolism, host–microbe interactions and genetic information processing. Representative enriched pathways included glycolysis, fructose and mannose metabolism, glycerolipid metabolism, NOD‐like receptor signalling, bacterial invasion of epithelial cells, extracellular matrix (ECM)‐receptor interaction, ribosome and RNA polymerase (Figure 5; Table S1).

Conversely, LG‐associated bacteriomes showed significant predicted enrichment of pathways involved in energy metabolism, lipid metabolism and amino acid metabolism. Representative pathways included pyruvate, propanoate and butanoate metabolism, steroid biosynthesis and arginine, proline and phenylalanine metabolism (Figure 5; Table S1).

Collectively, these findings demonstrate that bacterial communities associated with LG and HG PMP exhibit distinct predicted functional profiles, whereas no significant differences in predicted functional pathways were detected between control tissues and PMP samples.

## 4 Discussion

This study provides the first comprehensive multi‐kingdom characterisation of the microbiome associated with PMP by integrating bacterial and fungal metabarcoding with culture‐based microbiology, histochemical staining and RNAscope in situ hybridisation. Recent studies have established that many solid tumours harbour complex tumour‐associated microbial communities, highlighting the microbiome as an integral component of the tumour microenvironment (Nejman et al. 2020; Sepich‐Poore et al. 2021). To our knowledge, this is the first study demonstrating the presence of a fungal microbiome in PMP while providing complementary molecular, microbiological and spatial evidence supporting the persistence of bacterial and fungal communities within tumour‐associated mucin. Collectively, these findings expand the current biological landscape of PMP and support the concept that tumour‐associated mucin constitutes a previously unrecognised polymicrobial niche. Although PMP is an exceptionally rare malignancy, with an estimated incidence of only 2–4 cases per million inhabitants per year (Smeenk et al. 2008), the combination of multiple orthogonal methodologies provides a comprehensive characterisation of the tumour‐associated microbiome and substantially strengthens the biological relevance of the observations reported here.

Previous studies have consistently reported the presence of bacterial DNA and viable microorganisms in PMP‐associated mucin, supporting the concept that tumour‐associated mucin may provide a favourable niche for microbial persistence (Gilbreath et al. 2013; Nieciecki et al. 2025; Portela et al. 2025; Grant et al. 2026). Our findings are consistent with these observations, confirming the presence of bacterial communities in both human PMP samples and the corresponding PDX models. However, our study further demonstrates that these bacterial communities are not homogeneous across PMP. While bacterial signatures were also detected in control appendiceal tissues, consistent with the intestinal origin of most PMP cases (Khamzina et al. 2022; Villarejo‐Campos et al. 2023; Grant et al. 2026), bacterial community composition differed according to tumour grade, with HG tumours displaying compositional shifts and increased inter‐sample heterogeneity. These findings suggest that the biological relevance of the PMP‐associated bacteriome lies not simply in the presence of microorganisms, but in the progressive reorganisation of bacterial communities within the tumour‐associated mucin.

The most novel finding of this study is the identification of a fungal component within the PMP‐associated microbiome. While previous investigations of PMP have focused almost exclusively on bacterial communities (Gilbreath et al. 2013; Khamzina et al. 2022; Nieciecki et al. 2025; Portela et al. 2025), the fungal component had remained completely unexplored. Recent reviews have highlighted this lack of knowledge and identified characterisation of the microbial ecosystem, particularly, within the spatial context of the tumour microenvironment, as a major research priority in PMP (Grant et al. 2026). Using 18S rRNA gene sequencing, we detected fungal DNA in all study groups, with a higher fungal burden in tumour‐associated mucin than in control tissues. Importantly, fungal structures were independently confirmed by PAS and CFW staining, while RNAscope further demonstrated the spatial localisation of fungal 18S rRNA within epithelial and mucin‐rich tumour regions. Together, these complementary approaches provide convergent molecular, histological and spatial evidence supporting fungi as a genuine component of the PMP tumour microenvironment rather than an artefact of DNA‐based sequencing.

Our findings are consistent with the growing recognition that fungal communities constitute an integral component of the tumour microbiome across multiple human malignancies (Dohlman et al. 2022, 2025; Narunsky‐Haziza et al. 2022; Li et al. 2023; Cao et al. 2024; Wang et al. 2024). Tumour‐associated fungi have been reported across a broad range of malignancies, with accumulating evidence supporting cancer type‐specific fungal communities and potential interactions with bacterial and host components of the tumour microenvironment (Dohlman et al. 2022, 2025; Narunsky‐Haziza et al. 2022; Wang et al. 2024). Although the biological role of fungi in PMP remains unknown, the identification of a previously unrecognised tumour‐associated mycobiome opens new avenues for investigating multi‐kingdom microbial interactions within the mucin‐rich tumour microenvironment.

It is still unknown how microorganisms reach the mucin associated with the PMP and how they persist there. Given that most cases of PMP originate in the appendix, one plausible route is the local translocation of microorganisms from the intestinal or appendiceal microbiota following disruption of the appendiceal barrier and dissemination of mucinous tumour material into the peritoneal cavity. More broadly, microorganisms have been detected in tissues that were traditionally considered sterile and there is experimental evidence to suggest that gut bacteria can translocate via the mesenteric lymph nodes and spread to distant organs (Michán‐Doña et al. 2024; Xu et al. 2025). Whether similar mechanisms contribute to the bacterial and fungal communities detected in PMP remains to be established. Once microorganisms reach the peritoneal compartment, the abundant extracellular mucin and hypoxic microenvironment characteristic of PMP may provide a permissive ecological niche for their persistence. Future studies comparing intestinal, appendiceal and tumour‐associated microbial communities will be required to determine their origin and routes of dissemination.

An important strength of the present study is the integration of multiple independent methodologies to characterise the PMP‐associated microbiome. This multimodal strategy addresses one of the major challenges recently highlighted in PMP research: validating microbial localisation within the tumour microenvironment beyond sequencing‐based approaches (Grant et al. 2026). One of the major challenges in tumour microbiome research is distinguishing true tumour‐associated microorganisms from environmental contaminants or residual microbial DNA, particularly, in low‐biomass samples (Nejman et al. 2020; Cao et al. 2024). Rather than relying exclusively on sequencing‐based approaches, we combined bacterial and fungal metabarcoding with culture‐based microbiology, histochemical staining and RNAscope in situ hybridisation to evaluate microbial presence using complementary methodologies. Bacterial viability was confirmed by culture in the majority of PMP samples and in all analysed PDX models, while fungal structures were independently visualised by PAS and CFW staining. Furthermore, RNAscope demonstrated the spatial localisation of bacterial 16S and fungal 18S rRNA within epithelial and mucin‐rich tumour regions in both patient samples and PDX models. Although these complementary methodologies do not directly demonstrate microbial function in situ, together they provide substantially stronger evidence than sequencing alone for the persistence and spatial organisation of microbial communities within PMP mucin.

An additional finding of this study is that bacterial communities associated with LG and HG PMP exhibited distinct predicted functional profiles. Although no significant differences in predicted functional pathways were detected between control tissues and PMP samples, pathway enrichment analysis identified multiple functional categories that differed significantly between tumour grades. In particular, HG‐associated bacteriomes showed predicted enrichment of pathways related to carbohydrate metabolism, host–microbe interaction‐related pathways and genetic information processing, whereas LG‐associated bacteriomes were enriched in pathways involved in short‐chain fatty acid metabolism, lipid metabolism and amino acid metabolism. These observations indicate that bacterial communities associated with different histological grades exhibit distinct predicted functional profiles. However, because these analyses are inferred from 16S rRNA gene sequencing rather than direct measurements of microbial gene expression or metabolism, they should be interpreted with caution. Functional prediction approaches provide valuable biological hypotheses but require confirmation using shotgun metagenomics, metatranscriptomics or metabolomics to establish the functional activity of tumour‐associated microbial communities (Douglas et al. 2020).

### 4.1 Biotechnological Perspective

From a biotechnological perspective, PMP represents a challenging model for microbial investigation because of the physicochemical complexity of the mucin‐rich tumour matrix. The integrated workflow used here illustrates how complementary technologies can help overcome this challenge by combining microbial enrichment and metabarcoding with culture‐based identification, histochemical detection and spatial RNA hybridisation. Importantly, this strategy extends microbial profiling beyond sequencing alone by combining recovery of viable bacteria with complementary histological and spatial validation of microbial signals. The future incorporation of shotgun metagenomics, metatranscriptomics and metabolomics could provide strain‐level resolution and direct information on microbial functional activity, while emerging spatial and single‐cell technologies could help to unravel the interactions between microorganisms, mucin, tumour cells and host components. In this context, PMP‐derived experimental models could provide a platform for the functional validation of hypotheses generated by these multi‐omics approaches. These integrated biotechnology‐driven strategies could ultimately facilitate the transition from descriptive microbial profiling to mechanistic research into multi‐kingdom tumour ecosystems.

This study has several limitations that should be considered when interpreting the results. First, the cohort size is necessarily limited by the exceptional rarity of PMP, a malignancy with an estimated incidence of only 2–4 cases per million inhabitants per year (Smeenk et al. 2008). Nevertheless, the number of patients included is comparable to previous microbiome studies in PMP (Gilbreath et al. 2013; Khamzina et al. 2022; Nieciecki et al. 2025; Portela et al. 2025), while the present work substantially extends these reports by providing the first integrated multi‐kingdom characterisation of the PMP‐associated microbiome. Importantly, all samples were collected prospectively using a standardised protocol, processed under identical experimental conditions and classified according to the current PSOGI criteria, thereby minimising clinical and technical heterogeneity within the cohort. Second, all patients were recruited from a single tertiary referral centre. Although this approach may limit the generalizability of the findings, it also ensured consistency in patient management, sample collection and processing. Future multicentre studies including larger independent cohorts will be required to validate these observations. In addition, although PMP‐derived xenograft models provide a valuable platform for studying tumour‐associated microorganisms, their reliance on immunodeficient hosts limits investigation of tumour–microbe–immune interactions, highlighting the need for more physiologically relevant experimental models (Grant et al. 2026). Finally, the functional analyses were inferred from bacterial taxonomic profiles and therefore represent predicted rather than directly measured microbial functions. Future studies combining shotgun metagenomics, metatranscriptomics and metabolomics will be required to validate the functional significance of the microbial communities identified in PMP.

## 5 Conclusions

This study provides the first comprehensive multi‐kingdom characterisation of the microbiome associated with PMP, demonstrating that tumour‐associated mucin harbours both bacterial and fungal communities. By integrating bacterial and fungal sequencing with culture‐based microbiology, histochemical staining and RNAscope, we provide complementary evidence supporting the presence and spatial organisation of microorganisms within the PMP tumour microenvironment. Furthermore, bacterial communities associated with different histological grades exhibited distinct predicted functional profiles, highlighting grade‐associated differences in the predicted functional organisation of the PMP‐associated bacteriome. Collectively, these findings redefine PMP mucin as a polymicrobial niche and provide a foundation for future studies investigating the contribution of bacteria–fungi–host interactions to PMP biology and their potential diagnostic and therapeutic implications.

## Author Contributions

**Rafaela Pezzopane:** investigation, software, formal analysis, methodology, data curation, writing – original draft. **Marina Barbudo‐Lunar:** investigation, methodology, software, data curation, formal analysis, writing – original draft. **Florina I. Bura:** methodology, formal analysis. **Melissa Granados‐Rodríguez:** methodology, formal analysis. **Ana Martínez‐López:** methodology, formal analysis. **Lidia Rodríguez‐Ortiz:** methodology, formal analysis. **Blanca Rufián‐Andújar:** methodology. **Javier López‐Jiménez:** methodology. **Verónica Luna‐Guerrero:** methodology. **José Alhama:** writing – original draft, supervision, investigation. **Carmen Michán:** investigation, writing – original draft, supervision. **Mari C. Vázquez‐Borrego:** investigation, conceptualization, writing – original draft, writing – review and editing, software, formal analysis, supervision, funding acquisition. **Álvaro Arjona‐Sánchez:** conceptualization, funding acquisition, writing – original draft, writing – review and editing, project administration, supervision, investigation. **Antonio Romero‐Ruiz:** conceptualization, investigation, funding acquisition, writing – original draft, writing – review and editing, software, formal analysis, supervision.

## Funding

This work was supported by the following funding sources: Project “PI22/01213”, funded by Instituto de Salud Carlos III (ISCIII) and co‐funded by the European Union. Additional funding was provided by Instituto de Salud Carlos III (ProteoRed‐0000141), Fundación Científica Asociación Española Contra el Cáncer (PRYES223170ARJO), and Spanish Ministry of Universities (FPU2022‐04116 and FPU2023‐01856).

## Ethics Statement

Ethical standards of the Helsinki Declaration and the World Medical Association, with the approval of the University of Córdoba/IMIBIC and the research ethics committee of Córdoba (CETICO, *Comité de Ética de la Investigación de Córdoba*), can be implemented within the HURS and the IMIBIC (protocol code PI22/01213, version 1, dated 02 June 2023).

## Consent

Written informed consent was obtained from each patient.

## Conflicts of Interest

The authors declare no conflicts of interest.

## Supporting information


**Figure S1:** Hierarchical composition of bacterial communities across PMP samples. Krona charts representing the hierarchical taxonomic structure and relative abundance of bacterial communities in individual control (C1–C5), low‐grade (LG1–LG5) and high‐grade (HG1–HG5) samples based on 16S rRNA gene sequencing. Concentric rings represent successive taxonomic levels and sector size reflects the relative abundance of each taxon.
**Figure S2:** Representative culture of viable bacteria recovered from human PMP samples and corresponding patient‐derived xenograft (PDX) tumours. Representative isolates identified as *Sphingomonas paucimobilis, Bacillus amyloliquefaciens* and *Bacillus licheniformis* are shown. R1 and R2 correspond to independent replicate cultures, whereas CP indicates the negative control plate. Taxonomic identification was confirmed by MALDI‐TOF and/or 16S rRNA gene sequencing.
**Figure S3:** Hierarchical composition of fungal communities across PMP samples. Krona charts representing the hierarchical taxonomic structure and relative abundance of fungal communities in individual control (C1–C5), low‐grade (LG1–LG5) and high‐grade (HG1–HG5) samples based on 18S rRNA gene sequencing. Concentric rings represent successive taxonomic levels and sector size reflects the relative abundance of each taxon.
**Figure S4:** Low‐magnification detection of microbial rRNA in PMP tissues by RNAscope chromogenic in situ hybridization (RNAscope‐CISH). Formalin‐fixed paraffin‐embedded (FFPE) PMP tissue sections (4–5 μm) containing epithelial and mucin‐rich regions were analysed. Detection of bacterial 16S rRNA and fungal 18S rRNA was performed in samples from PMP patients (P1–P4) and in a PMP patient‐derived xenograft (PDX) mouse model (M). Red chromogenic puncta represent amplified bacterial or fungal rRNA signals. Microbial rRNA was detected in both epithelial and mucin‐rich tumour regions in human PMP samples and the corresponding PDX model. Sections were counterstained with haematoxylin and ammonia. Scale bar = 25 μm. Original magnification ×20.
**Table S1:** Predicted metabolic pathways based on Tax4Fun analysis.

## Data Availability

The datasets used and analysed in this study are available upon reasonable request from the corresponding authors (b72rorua@uco.es and z32vabom@uco.es). In addition, the datasets supporting the conclusions of this article are publicly available in the Zenodo repository (https://doi.org/10.5281/zenodo.19500974).
