# Supplementary material for: Integrated Microbial Profiling Identifies Bacterial and Fungal Communities in Pseudomyxoma Peritonei Mucin
Source: Microb Biotechnol. 2026 Nov 3;19(11):e70457. doi: 10.1111/1751-7915.70457 (PMC13729090; doi:10.1111/1751-7915.70457)
Supplement: Supplementary file 1 [file MBT2-19-e70457-s001.pdf]

Additional file 1

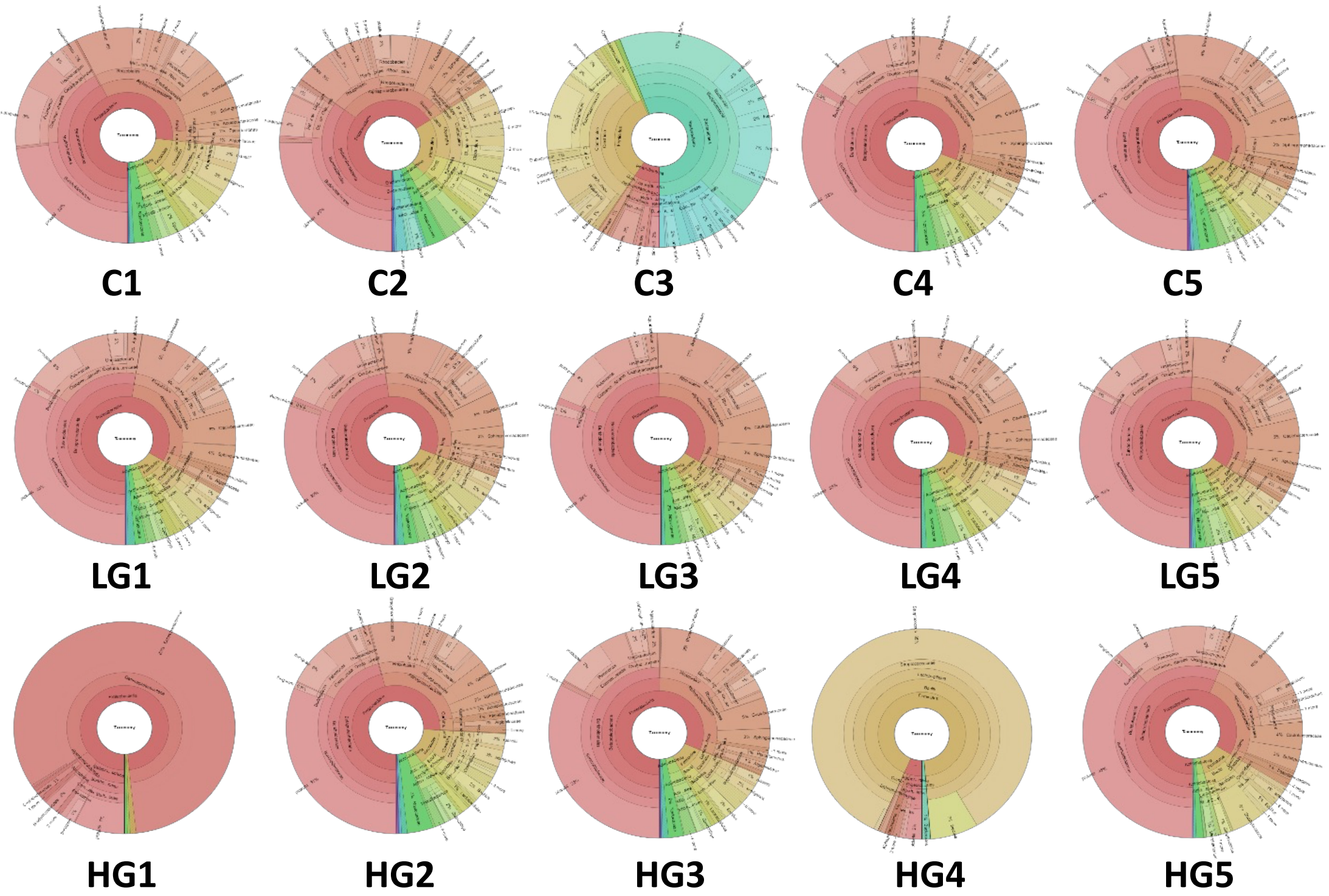

Fig. S1. Hierarchical composition of bacterial communities across PMP samples. Krona charts representing the hierarchical taxonomic structure and relative abundance of bacterial communities in individual control (C1–C5), low-grade (LG1–LG5), and high-grade (HG1–HG5) samples based on 16S rRNA gene sequencing. Concentric rings represent successive taxonomic levels, and sector size reflects the relative abundance of each taxon.

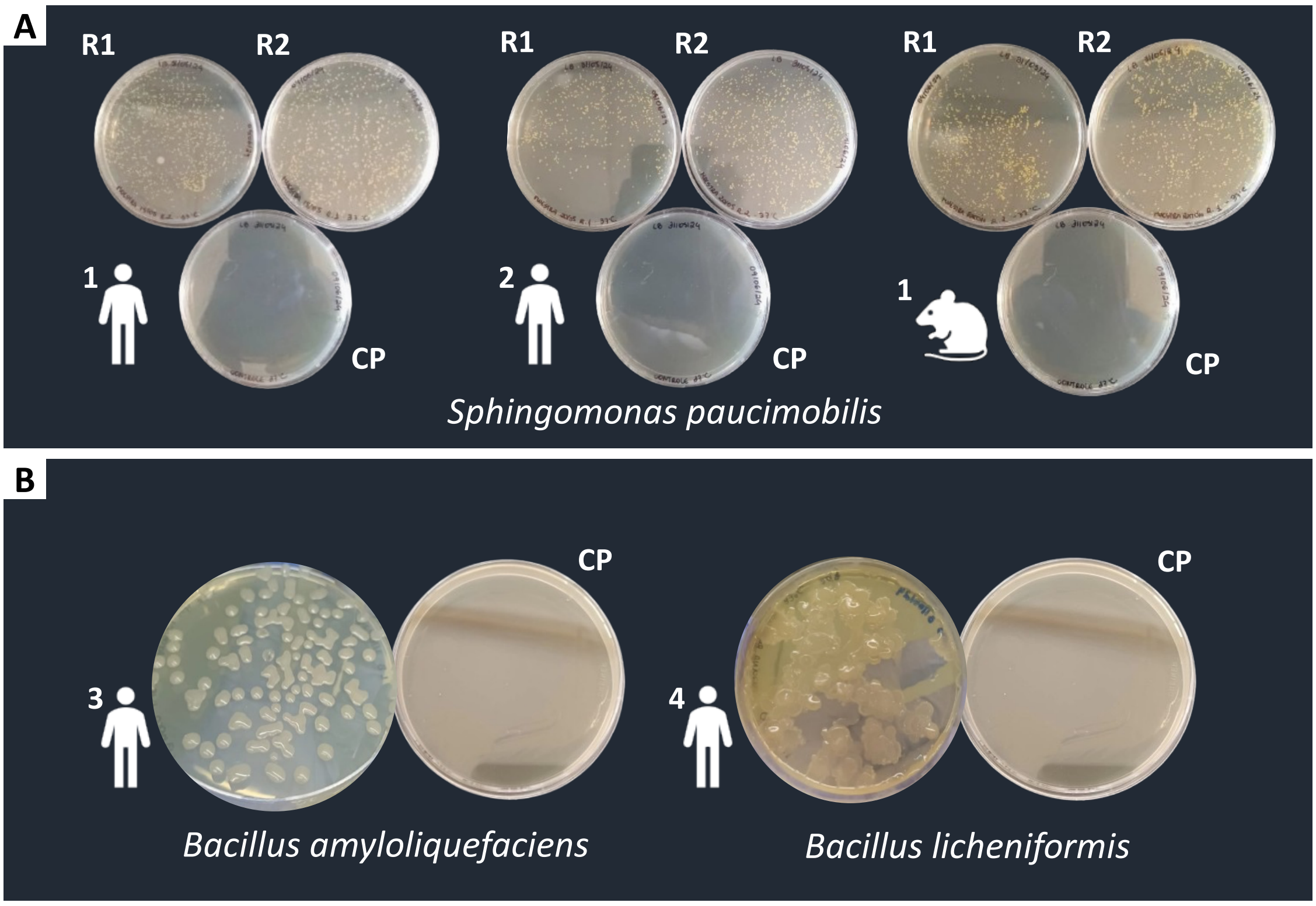

Fig. S2. Representative culture of viable bacteria recovered from human PMP samples and corresponding patient-derived xenograft (PDX) tumours. Representative isolates identified as *Sphingomonas paucimobilis*, *Bacillus amyloliquefaciens*, and *Bacillus licheniformis* are shown. R1 and R2 correspond to independent replicate cultures, whereas CP indicates the negative control plate. Taxonomic identification was confirmed by MALDI-TOF and/or 16S rRNA gene sequencing.

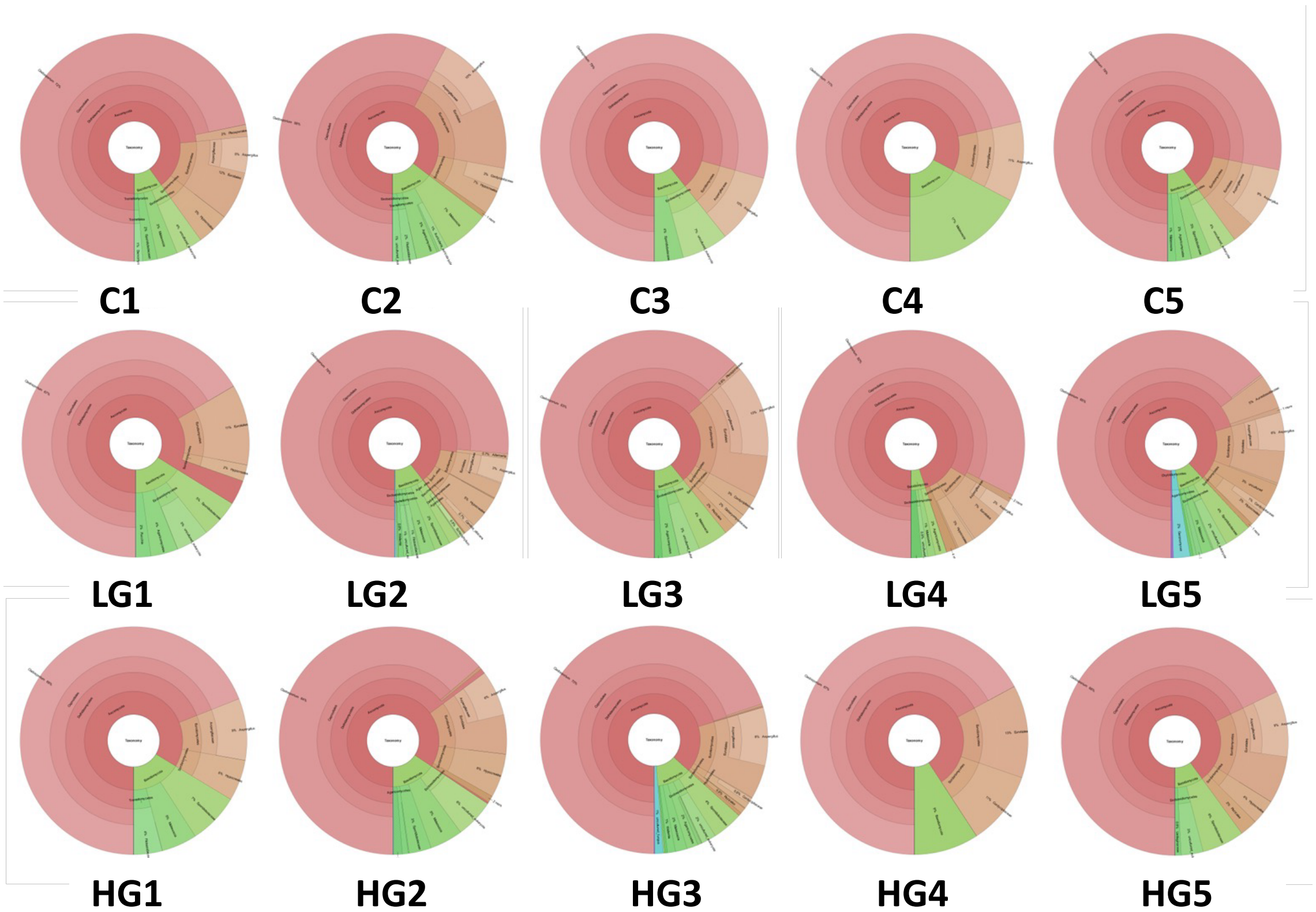

Fig. S3. Hierarchical composition of fungal communities across PMP samples. Krona charts representing the hierarchical taxonomic structure and relative abundance of fungal communities in individual control (C1–C5), low-grade (LG1–LG5), and high-grade (HG1–HG5) samples based on 18S rRNA gene sequencing. Concentric rings represent successive taxonomic levels, and sector size reflects the relative abundance of each taxon.

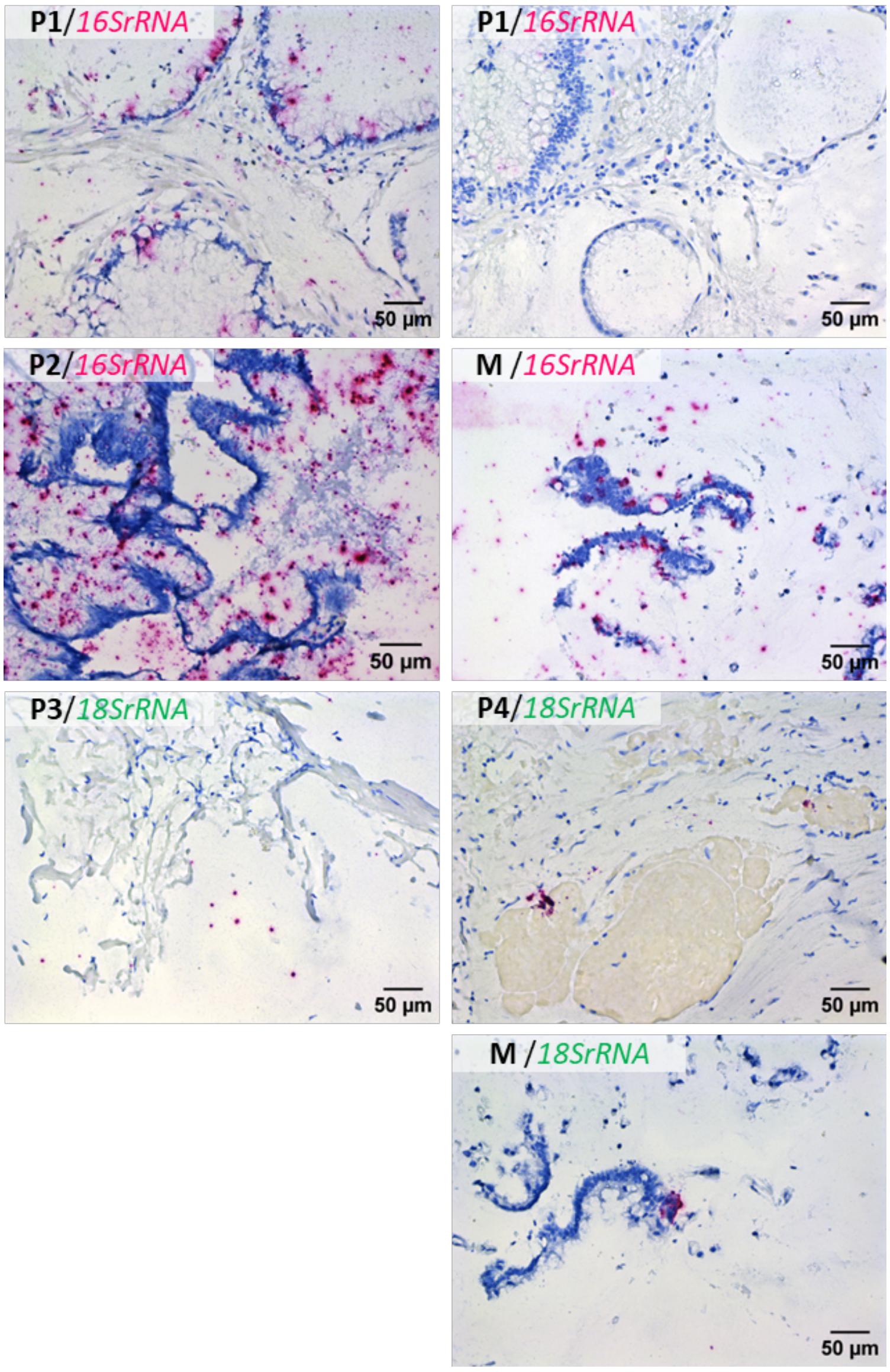

Fig. S4. Low-magnification detection of microbial rRNA in PMP tissues by RNAscope chromogenic in situ hybridization (RNAscope-CISH). Formalin-fixed paraffin-embedded (FFPE) PMP tissue sections (4–5 μm) containing epithelial and mucin-rich regions were analysed. Detection of bacterial 16S rRNA and fungal 18S rRNA was performed in samples from PMP patients (P1–P4) and in a PMP patient-derived xenograft (PDX) mouse model (M). Red chromogenic puncta represent amplified bacterial or fungal rRNA signals. Microbial rRNA was detected in both epithelial and mucin-rich tumour regions in human PMP samples and the corresponding PDX model.

Sections were counterstained with hematoxylin and ammonia. Scale bar = 25 μm. Original magnification ×20.

Table S1. Predicted metabolic pathways based on Tax4Fun analysis.

KEGG PATHWAY	METABOLIC PATHWAY	LG vs. C	HG vs. C	HG vs. LG
Amino acid metabolism	Arginine and proline metabolism			#
	Phenylalanine metabolism			#
Carbohydrate metabolism	Glycolysis / Gluconeogenesis			*
	Fructose and mannose metabolism			*
	Starch and sucrose metabolism			*
	Pyruvate metabolism			#
	Glyoxylate and dicarboxylate metabolism			#
	Propanoate metabolism			#
	Butanoate metabolism			#
Cellular community - eukaryotes	Focal adhesion			*
Circulatory system	Cardiac muscle contraction			#
D-Aminoacid metabolism	D-Alanine metabolism			*
Digestive system	Carbohydrate digestion and absorption			*
Endocrine and metabolic disease	Type II diabetes mellitus			*
Glycan biosynthesis and metabolism	Peptidoglycan biosynthesis			*
Immune system	NOD-like receptor signaling pathway			*
Infectious disease: bacterial	Bacterial invasion of epithelial cells			*
Lipid metabolism	Steroid biosynthesis			#
	Steroid hormone biosynthesis			#
	Glycerolipid metabolism			*
	Glycerophospholipid metabolism			*
	Biosynthesis of unsaturated fatty acids			#
Metabolism of cofactors and vitamins	Lipoic acid metabolism			*
Metabolism of other amino acids	beta-Alanine metabolism			#
	Taurine and hypotaurine metabolism			#
	Phosphonate and phosphinate metabolism			#
	Selenocompound metabolism			*
Metabolism of terpenoids and polyketides	Geraniol degradation			#
	Polyketide sugar unit biosynthesis		#	
	Biosynthesis of type II polyketide products			#
Neurodegenerative disease	Parkinsons disease			#
Nucleotide metabolism	Purine metabolism			*
	Pyrimidine metabolism			*
Replication and repair	Mismatch repair			*
	Homologous recombination			*
Signal transduction	Two-component system			#
Signaling molecules and interaction	ECM-receptor interaction			*

Transcription	RNA polymerase	*
Translation	Aminoacyl-tRNA biosynthesis	*
	Ribosome	*
Xenobiotics biodegradation and metabolism	Benzoate degradation	#
	Naphthalene degradation	#
	Drug metabolism - other enzymes	*

Metabolic pathways showing significant differences in abundance are highlighted. Green indicates pathways enriched in the reference group (Control for LG vs. C and HG vs. C comparisons; LG for HG vs. LG comparisons), whereas red indicates pathways enriched in the comparison group. For the HG vs. LG comparison, * indicates pathways significantly enriched in HG and # indicates pathways significantly enriched in LG. Statistical significance was defined as an adjusted $p < 0.05$ (Benjamini–Hochberg correction).
